# Colorectal cancer in patients of advanced age is associated with increased incidence of *BRAF* p.V600E mutation and mismatch repair deficiency

**DOI:** 10.3389/fonc.2023.1193259

**Published:** 2023-06-07

**Authors:** Eric S. Christenson, Hua-Ling Tsai, Dung T. Le, Elizabeth M. Jaffee, Jonathan Dudley, Rena R. Xian, Christopher D. Gocke, James R. Eshleman, Ming-Tseh Lin

**Affiliations:** ^1^ Department of Oncology, Sidney Kimmel Comprehensive Cancer Center at Johns Hopkins, Baltimore, MD, United States; ^2^ The Cancer Convergence Institute at Johns Hopkins, Johns Hopkins University School of Medicine, Baltimore, MD, United States; ^3^ Division of Quantitative Sciences, Johns Hopkins University, Baltimore, MD, United States; ^4^ Department of Pathology, Johns Hopkins University, Baltimore, MD, United States

**Keywords:** mismatch repair (MMR) deficiency, colorectal cancer, BRAF mutation V600E, late onset colorectal cancer, APC mutations

## Abstract

**Introduction:**

The highest incidence of colorectal cancer (CRC) is in patients diagnosed at 80 years or older highlighting a need for understanding the clinical and molecular features of these tumors. Methods. In this retrospective cohort study, 544 CRCs underwent next generation sequencing and mismatch repair (MMR) evaluation. Molecular and clinical features were compared between 251 patients with traditional-onset CRC (50-69 years at diagnosis) and 60 with late-onset CRC (>80 years at diagnosis).

**Results:**

Late-onset CRC showed a significantly higher rate of right-sided tumors (82% vs 35%), MMR deficiency (35% vs. 8%) and *BRAF* p.V600E mutations (35% vs. 8%) and a significantly lower rate of stage IV disease (15% vs 28%) and *APC* mutations (52% vs. 78%). Association of these features with advanced age was supported by stratifying patients into 6 age groups (<40, 40-49, 50-59, 60-69, 70-79 and >80 years). However, the age-related rise in MMR deficient (dMMR) CRC was only seen in the female patients with an incidence of 48% (vs. 10% in the male patient) in the >80y group. In addition, *BRAF* p.V600E was significantly enriched in MMR deficient CRC of advanced age (67% in late-onset CRC). Categorizing CRC by mutational profiling, late-onset CRC revealed a significantly higher rate of dMMR/*BRAF* ^+^ *APC* ^-^ (18% vs. 2.0%), dMMR/*BRAF* ^-^ *APC* ^-^ (8.3% vs. 1.2%) and MMR proficient (pMMR)/*BRAF* ^+^ *APC* ^-^ (12% vs. 4.0%) as compared to traditional-onset CRC.

**Discussion:**

In summary, there was a higher rate of dMMR and *BRAF* p.V600E in late-onset CRC, independently or in combination. The higher incidence of dMMR in late-onset CRC in females is most likely predominantly driven by *BRAF* p.V600E induced hypermethylation. Prospective studies with treatment plans designed specifically for these older patients are warranted to improve their outcomes.

## Introduction

While important progress has been made in the treatment of colorectal cancer (CRC) over the last decade, CRC still represents the second leading cause of cancer related death in the United States (1, 2). Recently, a disturbing rise in CRC incidence amongst young-adults has increased emphasis on understanding age-related differences in clinical presentation and molecular features of CRC (3). Investigations in this area have primarily focused on exploring the differences between these early-onset CRC and those that occur in individuals diagnosed at age 50 and above. However, the highest incidence of CRC is seen in patients at age 80 and older highlighting the importance of also understanding how tumors that occur in this age group might differ from the general population (2).

Several prior groups have sought to address this question with some cohorts reporting *BRAF* and *KRAS* mutations to be more common in CRC seen in patients with later age of diagnosis which is particularly relevant given the implications of these mutations for targeted therapies (4–8). Several of these cohorts additionally suggested that this higher rate of *BRAF* mutations translated into an elevated rate of mismatch repair deficient (dMMR) disease while another did not show any increase in dMMR disease (9–11). Given the higher risk and toxicity with surgery and chemotherapy in patients of advanced age, understanding the prevalence of targetable and/or prognostic genomic markers, such as *KRAS*, *NRAS* and *BRAF* p.V600E mutations and MMR deficiency is critical to guide management (12, 13).

## Materials and methods

To investigate how age impacts the clinical and molecular features of CRC in the Johns Hopkins University (JHU) catchment area, all CRC tumors which underwent molecular testing following the launch of our solid tumor next-generation sequencing (NGS) panel in September 2017 through January 2020 were systematically collected along with the patient’s clinical data. The Johns Hopkins Medicine institutional review board granted approval to this study.

### Clinical features

Patient’s age at diagnosis and gender as well as other clinical features of the tumor were recorded. These included primary site of disease, histologic grade and TNM staging as determined by the seventh or eighth edition of the American Joint Committee on Cancer staging system as recommended at the time of pathologic evaluation.

### Mismatch repair deficiency

MMR status was determined by using immunohistochemistry (IHC) stains and/or microsatellite instability assay. IHC results for MMR proteins (MLH1, MSH2, MSH and PMS2) were obtained from surgical pathology reports. Microsatellite stability assay was determined by comparing 5 mononucleotide microsatellite loci between neoplastic and nonneoplastic tissues using the MSI Analysis System Version 1.2 (Promega, Madison, WI) as described previously (14).

### Molecular testing by next generation sequencing

All patients underwent testing on an internal solid tumor NGS panel as described previously (15–17). Briefly, DNA was extracted from formalin fixed, paraffin embedded tissues using the Tissue Preparation System (Siemens, Berlin, German), and measured by Qubit 2.0 Fluorometer (Life Technologies, Carlsbad, California). Libraries were prepared using the SureSelect-XT Target Enrichment System (Agilent Technologies Santa Clara, CA) and an Agilent custom panel covering full coding regions of over 400 cancer-associated genes (https://pathology.jhu.edu/jhml-services/assets/test-directory/SolidTumorPanel-II_GeneList_v5.0). NGS was performed to an average 500-1000x read depth by the HiSeq 2500 or NovaSeq 6000 system platform using Illumina paired end technology (Illumina, San Diego, CA, USA). All reads were aligned to human reference sequence genome assembly hg19 (NCBI build GRCh37) using the Burrows-Wheeler alignment (BWA) algorithm. BMA files were generated using Picard Tools v1.119 and variant calling was performed using an in-house variant caller algorithm as well as a third-party variant caller (Haplotyper Genome Analysis TK-3.3). Variants were reviewed using the Integrated Genomics Viewer (Broad Institute, Cambridge, MA) and annotated utilizing the COSMIC, dbSNP and Annovar databases (18). The limit of detection was 5% mutant allele. A panel of hotspot mutations was reviewed using the Integrated Genomics Viewer at a threshold of 2% mutant allele. This included codons 12, 13, 59, 61, 117 and 146 of the KRAS and NRAS gene and codon 600 of the BRAF gene. The assay was validated for clinical reporting at a Clinical Laboratory Improvement Amendments (CLIA) certified clinical laboratory.

To minimize multiple comparisons, molecular markers analyzed in the current study included only those recommended as standard-of-care (*BRAF*, *KRAS*, *NRAS* mutations and MMR deficiency) according to the guidelines from the American Society for Clinical Pathology, College of American Pathologists, Association for Molecular Pathology and the American Society of Clinical Oncology and those with a higher incidence (>20%) of mutations in CRC (*APC*, *PIK3CA* and *TP53*) (12, 19).

### Statistical analysis

To determine whether patients with advanced age (80 years or older) at the time of diagnosis of CRC had molecularly and clinically distinct tumors, we compared this cohort of patients which we referred to as late-onset (LO) disease, to patients diagnosed with CRC from 50 to 69 years of age which we referred to as traditional-onset (TO) disease *via* Fisher exact testing or Chi-square testing.

In addition, the age-related changes on the molecular features across the entire cohort were assessed by stratification of age at the time of diagnosis as 6 groups (<40, 40-49, 50-59, 60-69, 70-79, and 80+), or by age at the time of diagnosis as a continuous variable. Patient characteristic and mutation proportion differences across age cohorts were evaluated *via* Chi-square testing.

Mutation profile at a patient level was presented with the correlations between mutations estimated *via* Cramér’s phi coefficient and tested *via* Pearson’s Chi-square test. Model based regression analyses were conducted *via* logistic regression with two‐way interaction effects between patient characteristics and age in relation to mutations examined. Non-linear relationships between age and mutations were also evaluated. In the case of significant interactions between age and mutations, the regression models were stratified by the corresponding variable (gender in our study). In the case of significant non-linear associations between age and mutations, age was categorized into three groups (≤ 49, 50-69, ≥ 70). Otherwise, the association between age and mutations was assessed by increasing decade of age at diagnosis. Multivariable analysis (MVA) was conducted with adjustments selected based on testing results between age cohorts and patient characteristics. *P*-values of 0.05 or lower were considered statistically significant without multiplicity adjustment. Analyses were compiled in Microsoft excel (Microsoft, Redmond, USA) and R version 4.2.2 (R Foundation for Statistical Computing, Vienna, Austria).

## Results

### Association of aging with clinical and molecular features

A total of 566 CRC specimens were analyzed using the solid tumor NGS panel. Twenty-two patients did not have corresponding MMR results and were excluded from statistical evaluation. Of these 22 patients, 9 were from the late-onset CRC (≥80y) representing 13% (9/69) of this population compared to 0%-2.5% of the other age groups (*P* = 0.001). Among the late-onset CRC with no known MMR status, wild-type *APC* was seen in 5 of 9 patients, including 3 patients with *BRAF* p.V600E.

**Figure 1:**
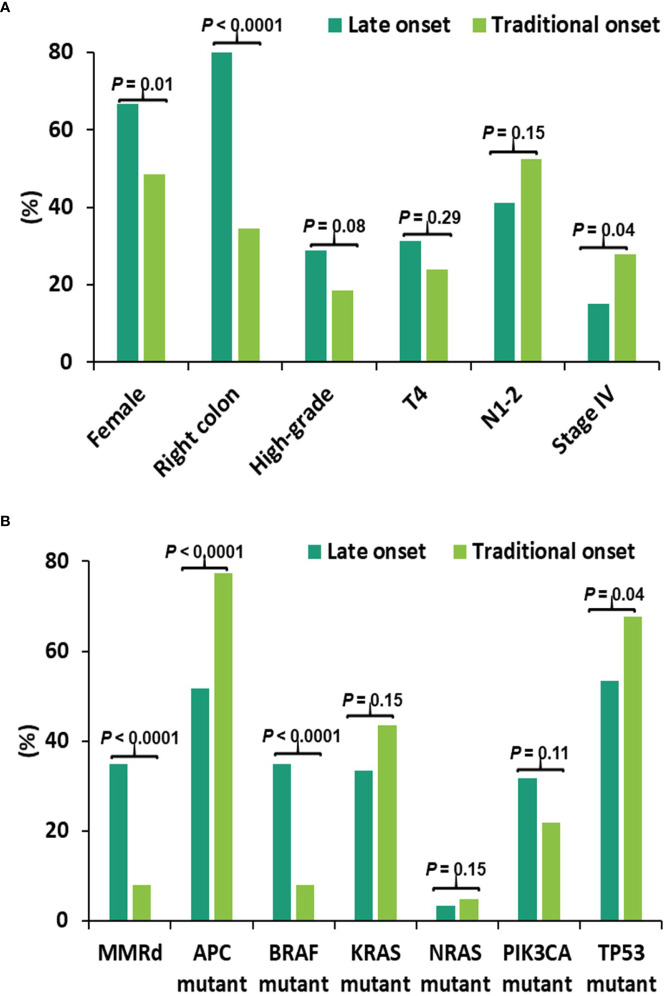
Clinical and molecular features in late-onset CRC (≥80y, n = 60) and traditional-onset CRC (50-69y, n = 251). **(A)** Clinical Features. High-grade: histomorphology of the primary tumor. T4, N1-2 and stage IV: TNM staging at diagnosis. Late-onset CRC showed a significantly higher rate of female gender and primary CRCs within right colon and a significantly lower incidence of stage IV disease at presentation. **(B)** Molecular Features. Late-onset CRC showed a significantly higher rate of mismatch repair deficiency (dMMR) and *BRAF* p.V600E mutation (*BRAF* mutant) and a significantly lower rate of *APC* and *TP53* mutations.

CRC patients (n = 544) with NGS and MMR results were compared between late-onset (LO) CRC arising in older individuals (80 years and older) and traditional-onset (TO) CRC occurring between 50 to 69 years of age. In late-onset CRC, there was a significantly higher rate of female gender (LO: 67% vs. TO: 49%), right-sided primary CRCs (LO: 82% vs. TO: 35%) and a lower rate of stage IV disease at diagnosis (LO: 15%, TO: 28%) and a trend of a higher rate of high-grade histomorphology (LO:29% vs. TO:17%) (**Figure 1A**).

Molecular features between these two age cohorts were also compared, identifying a higher rate of MMR deficiency (LO: 35% vs. TO: 8.0%) and *BRAF* p.V600E mutation (LO: 35% vs. TO: 8.0%), and a lower rate of *APC* (LO: 52% vs. TO: 78%) and *TP53* mutations (LO: 53% vs. TO: 68%) in late-onset CRC (**Figure 1B**).

**Figure 2:**
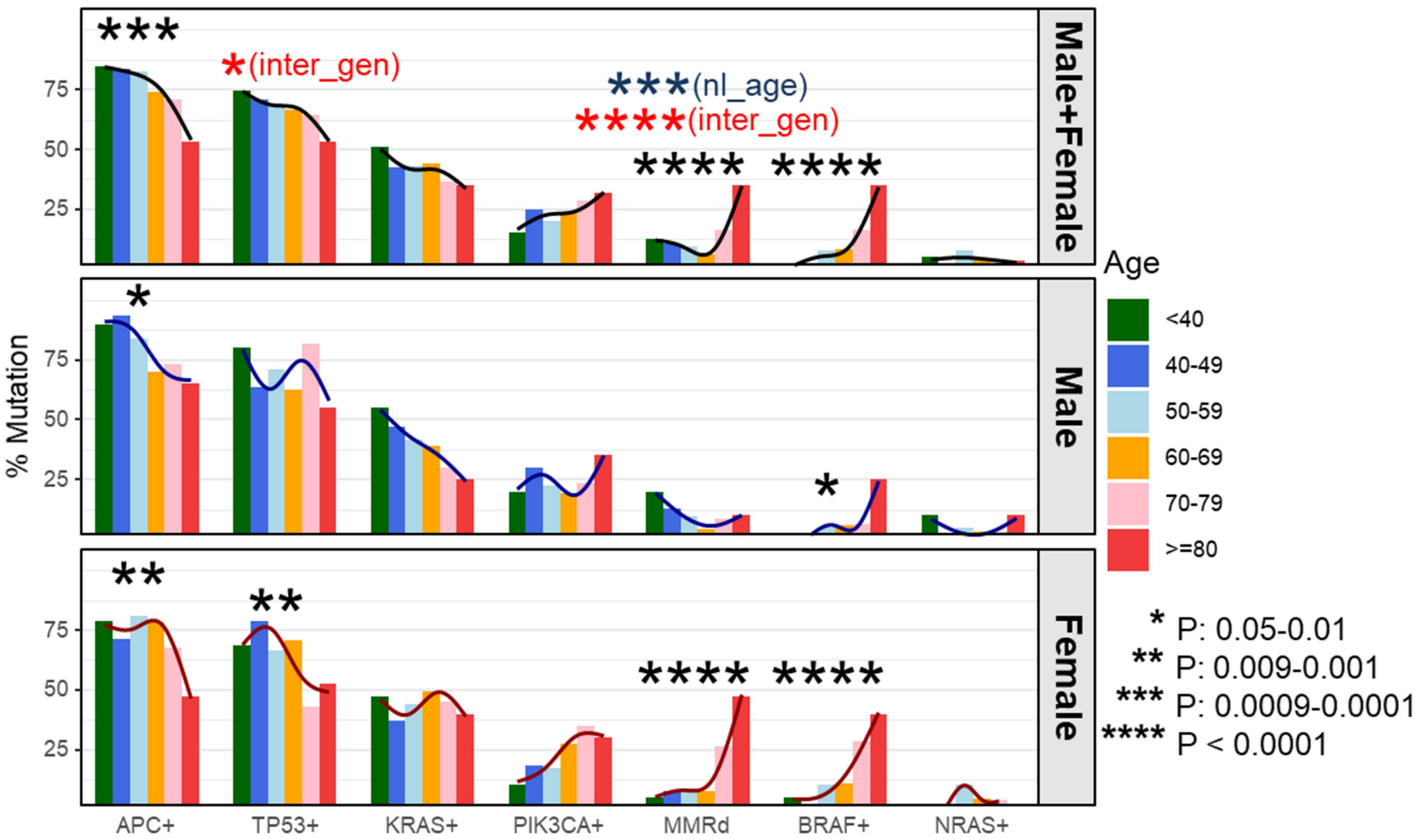
Percentage of dMMR/mutation by age cohorts and stratified by gender. We compared the incidence of each molecular alteration stratified by patient sex and decade of colorectal (CRC) diagnosis. This identified a negative correlation between incidence of *APC* mutations and age of CRC diagnosis in both male and female patients whereas a decreased incidence of *TP53* mutations was only seen in female patients. In contrast, the incidence of dMMR disease and BRAF p.V600E increased with patient age at CRC diagnosis. Stratifying by patient sex, both male and female patients had a rise in *BRAF* p.V600E incidence with increasing age whereas the increase in dMMR disease was confined to female patients. Black curve represents a smoothing trend by age cohorts at the corresponding mutation. dMMR - mismatch repair deficiency; nl_age - testing result in the entire cohort for nonlinearity effect of age at the corresponding mutation; inter_gen - testing result in the entire cohort for interaction effect between age and gender at the corresponding mutation.

To determine whether these associations were discrete changes once patients reached a particular age of diagnosis or represented a continuum of clinical characteristics and molecular changes, we stratified patients according to the decade of life in which CRC had been diagnosed and patient sex. Association with clinical and molecular features was tested across each age cohort (**Figure 2**). This investigation revealed that there was an age-related rise in dMMR CRC only in female patients while an increase in *BRAF* p.V600E in older patients was present in both male and female patients. In contrast, *APC* mutations decreased with age in both male and female patients. In the 80y group, dMMR was detected in 19 (48%) of 40 female patients and in 2 (10%) of 20 male patients (*P* = 0.004), while *BRAF* p.V600E was seen in 16 (40%) female patients and 5 (25%) male patients (*P* = 0.39). Aging was significantly associated with primary CRCs within right colon, absence of regional nodal metastasis (N0) and absence of systemic metastasis at diagnosis (stage I-III) (**Table 1**) as well as MMR deficiency, *BRAF* p.V600E mutation and wild-type *APC* (**Table 2**).

**Table 1:** Association of advanced age with clinical features.

	Total (n=544)	>80 y^a^ (n=60)	70-79 y^a^ (n=109)	60-69 y^a^ (n=132)	50-59 y^a^ (n=119)	40-49 y^a^ (n=85)	<40 y^a^ (n=39)	*P* value
Male	276 (51%)	20 (33%)	60 (55%)	67 (51%)	62 (52%)	47 (55%)	20 (51%)	0.11
Female	268 (49%)	40 (67%)	49 (45%)	65 (49%)	57 (48%)	38 (45%)	19 (49%)	
African-American	101 (19%)	10 (17%)	16 (15%)	25 (19%)	30 (25%)	13 (15%)	7 (18%)	0.40
Caucasian	368 (68%)	43 (72%)	82 (75%)	94 (71%)	74 (62%)	49 (58%)	26 (67%)	
Other/Unknown	75 (14%)	7 (12%)	11 (10%)	13 (10%)	15 (13%)	23 (27%)	6 (15%)	
Right	215 (40%)	49 (82%)	50 (46%)	41 (32%)	44 (37%)	23 (27%)	8 (21%)	10^-11^
Left	323 (60%)	11 (18%)	59 (54%)	87 (68%)	74 (63%)	62 (73%)	30 (79%)	
NK/NA	6	0	0	4	1	0	1	
LG^b^	403 (79%)	42 (71%)	83 (78%)	103 (82%)	87 (81%)	62 (78%)	26 (79%)	0.66
HG^b^	107 (21%)	17 (29%)	23 (22%)	22 (18%)	21 (19%)	17 (22%)	7 (21%)	
NK/NA	34	1	3	7	11	6	6	
T1-3^c^	295 (75%)	35 (69%)	66 (74%)	73 (78%)	60 (74%)	45 (80%)	16 (64%)	0.56
T4^c^	101 (25%)	16 (31%)	23 (26%)	21 (22%)	21 (26%)	11 (20%)	9 (36%)	
NK/NA	148	9	20	38	38	29	14	
N0^c^	186 (47%)	30 (59%)	47 (53%)	53 (56%)	30 (37%)	18 (32%)	8 (32%)	0.003
N1-2^c^	210 (53%)	21 (41%)	42 (47%)	41 (44%)	51 (63%)	38 (68%)	17 (68%)	
NK/NA	148	9	20	38	38	29	14	
Stage I-III^a^	389 (75%)	51 (85%)	85 (83%)	96 (76%)	77 (68%)	55 (67%)	25 (64%)	0.01
Stage IV^a^	135 (25%)	9 (15%)	18 (17%)	30 (24%)	37 (32%)	27 (33%)	14 (36%)	
NK/NA	20	0	6	6	5	3	0	

Right, right colon; left, left colon; LG, low-grade; HG, high-grade; NK/NA, not known/not applicable.

a At diagnosis of colorectal cancers.

b The histological grading was not evaluated in metastatic specimens.

c Pathological staging (TNM) after neoadjuvant chemoradiotherapy was not included.

**Table 2:** Association of advanced age with mutational profiling.

	Total	>80 y^a^	70-79 y^a^	60-69 y^a^	50-59 y^a^	40-49 y^a^	<40 y^a^	
	(n=544)	(n=60)	(n=109)	(n=132)	(n=119)	(n=85)	(n=39)	*P* value
dMMR	73 (13%)	21 (35%)	18 (17%)	8 (6.1%)	12 (10%)	9 (11%)	5 (13%)	4x10^-6^
pMMR	471 (87%)	39 (65%)	91 (83%)	124 (94%)	107 (90%)	76 (89%)	34 (87%)	
*APC* ^+^	398 (73%)	31 (52%)	75 (69%)	97 (73%)	97 (82%)	66 (78%)	32 (82%)	6x10^-4^
*APC* ^-^	146 (27%)	29 (48%)	34 (31%)	35 (27%)	22 (18%)	19 (22%)	7 (18%)	
*BRAF* ^+^	62 (11%)	21 (35%)	18 (17%)	11 (8.3%)	9 (7.6%)	2 (2.4%)	1 (2.6%)	2x10^-9^
*BRAF* ^-^	482 (89%)	39 (65%)	91 (83%)	121 (92%)	110 (92%)	83 (98%)	38 (97%)	
*KRAS* ^+^	222 (41%)	20 (33%)	39 (36%)	58 (44%)	51 (43%)	34 (40%)	20 (51%)	0.40
KRAS^-^	322 (59%)	40 (67%)	70 (64%)	74 (56%)	68 (57%)	51 (60%)	19 (49%)	
*NRAS* ^+^	21 (3.9%)	2 (3.3%)	4 (3.7%)	3 (2.3%)	9 (7.6%)	2 (2.4%)	1 (2.6%)	0.30
*NRAS* ^-^	523 (96%)	58 (97%)	105 (96%)	129 (98%)	110 (92%)	83 (98%)	38 (7%)	
*PIK3CA* ^+^	132 (24%)	19 (32%)	31 (28%)	31 (23%)	24 (20%)	21 (25%)	6 (15%)	0.34
*PIK3CA* ^-^	412 (76%)	41 (68%)	78 (72%)	101 (77%)	95 (80%)	64 (75%)	33 (85%)	
*TP53* ^+^	361 (66%)	32 (53%)	70 (64%)	88 (67%)	82 (69%)	60 (71%)	29 (74%)	0.23
*TP53* ^-^	183 (34%)	28 (47%)	39 (36%)	44 (33%)	37 (31%)	25 (29%)	10 (26%)	

BRAF^+^, BRAF p.V600E mutation; dMMR, mismatch repair deficiency; pMMR, mismatch repair proficiency.

a At diagnosis of colorectal cancers.

### Association with dMMR, *BRAF* p.V600E and wild-type *APC*

**Figure 3:**
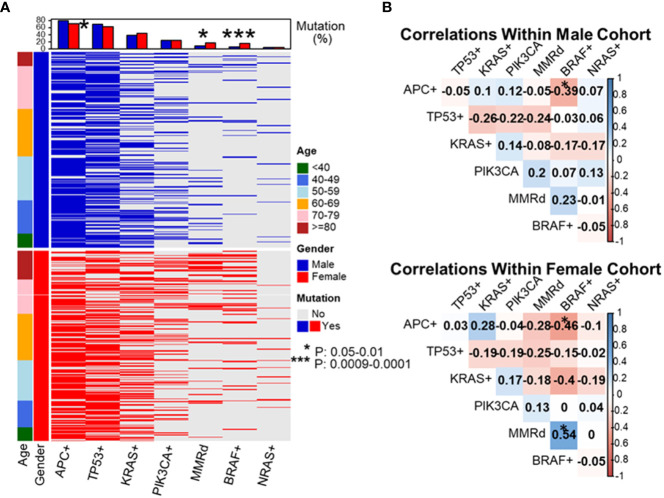
Evaluation of molecular features of patient tumors by decade of diagnosis. To dissect which genetic alterations tend to co-segregate or be mutual exclusive as a function of age and gender, we created a mutational profile of our patient cohort separated by gender and listing patients in ascending order of age at diagnosis **(A)**. We then performed pairwise correlation coefficients by gender looking at the association between each individual molecular alteration and the others listed **(B)**. These data revealed *APC*+ was associated with a lower rate of *BRAF*+ disease in both male and female patients. BRAF+ disease was associated with a statistically higher rate of mismatch repair deficiency (dMMR) in female patients and a trend towards higher rate of *BRAF*+ disease in dMMR colorectal cancer in male patients. *P-value: 0.05-0.01, ***P-value: 0.0009-0.0001.

As shown in previous cohort studies, dMMR CRCs demonstrated a significantly higher incidence of *BRAF* p.V600E (48% vs. 5.7%, *P* < 0.0001) and wild-type *APC* (56% vs. 22%, *P* < 0.0001) as compared to pMMR CRCs in the current cohort (20). To assess whether this elevated rate of *BRAF* p.V600E mutations might be responsible for the increase in dMMR disease amongst the late-onset CRC population, the co-occurrence between *BRAF* p.V600E mutations and MMR deficiency was assessed. The proportion of dMMR tumors with *BRAF* p.V600E was enriched with advanced age (67% in 80y group and 70-79y group, 63% in 60-69y group, 25% in 50-59y group, 11% in 40-49y group and 0% in <40y group, *P* = 0.003). In the overall cohort, *BRAF* p.V600E mutated CRC showed a significantly higher incidence of MMR deficiency compared to CRC without *BRAF* p.V600E (56% vs. 7.9%, *P* < 0.0001). *BRAF* p.V600E tumors also exhibited a lower rate of *APC* mutations (16% vs. 80%, *P* < 0.0001) demonstrating a strong co-segregation among these three variables (**Figure 3**).

As MMR deficiency, *BRAF* p.V600E, and wild-type *APC* CRC were all noted to increase in incidence as patients got older at their age of diagnosis, the relationship between these 3 variables was simultaneously assessed. *APC* mutational status was found to be strongly affected by the presence of *BRAF* p.V600E. *APC* and *BRAF* p.V600E mutations were nearly mutually exclusive in pMMR CRC (3/471, 0.6%), though there was a significant higher rate of co-existence of *APC* and *BRAF* p.V600E mutations in dMMR CRC (8 in 73, 11%, *P* < 0.0001).

**Figure 4:**
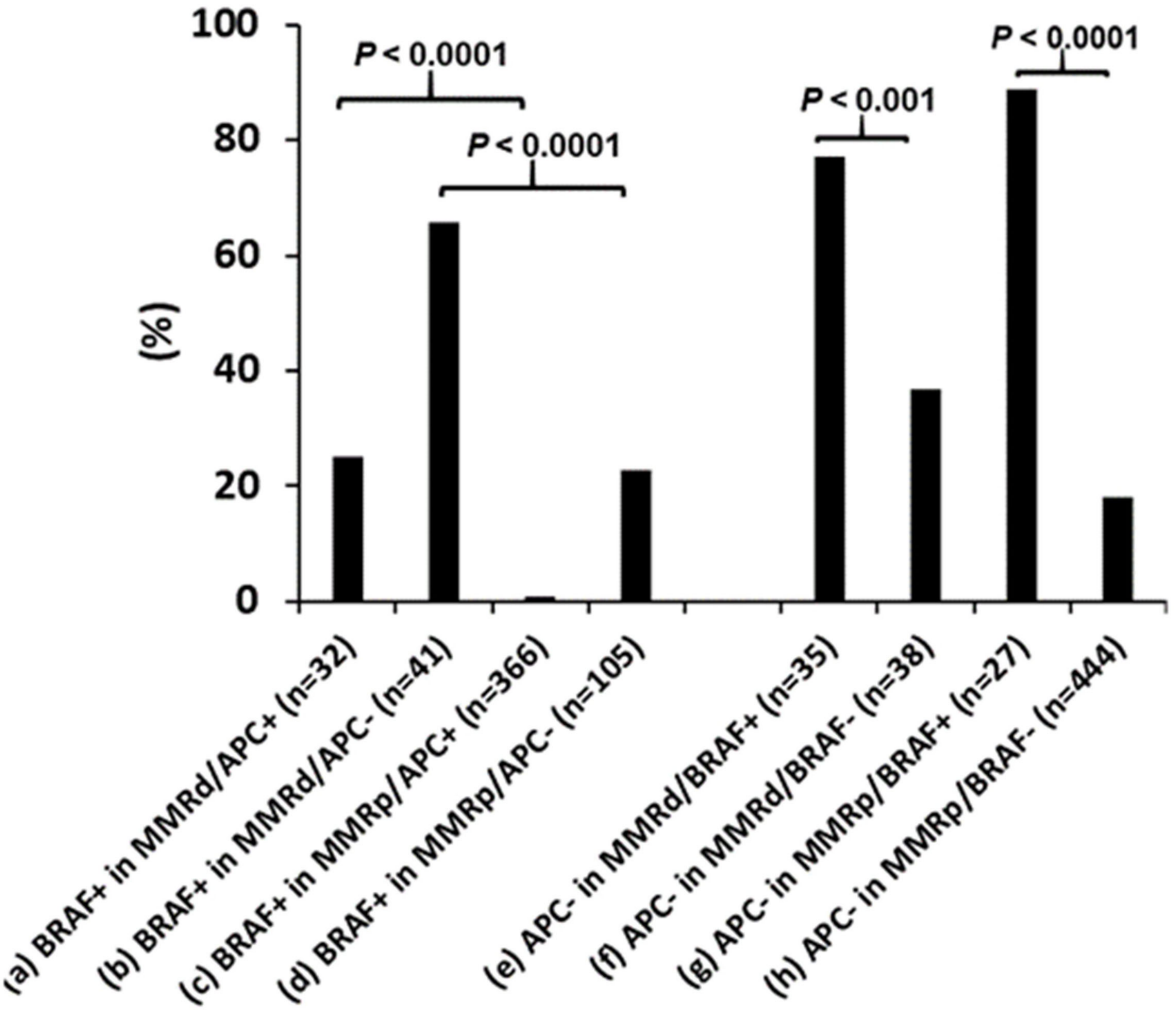
Co-existence of mismatch repair deficiency (dMMR), *BRAF* p.V600E and *APC* mutations. *BRAF* p.V600E (*BRAF*+) association with dMMR is independent of *APC* mutations and the *BRAF* p.V600E association with wild-type *APC* is independent of MMR status. pMMR: mismatch repair proficiency.

In contrast *BRAF* p.V600E was seen at a significant higher rate in dMMR CRCs with either mutant *APC* (25% vs. 0.8%, **Figure 4**) or wild-type *APC* (65% vs. 23%, **Figure 4**), indicating the association of *BRAF* p.V600E with dMMR is independent of *APC* mutational status. Wild-type *APC* was seen in a significantly higher incidence in *BRAF* p.V600E mutated CRCs with either deficient (77% vs. 37%, **Figure 4**) or proficient MMR (89% vs. 18%, **Figure 4**), indicating the association of *BRAF* p.V600E with wild-type *APC* is independent from MMR status.

### Categorization of CRC according to mutational profiling and its association with aging

**Figure 5:**
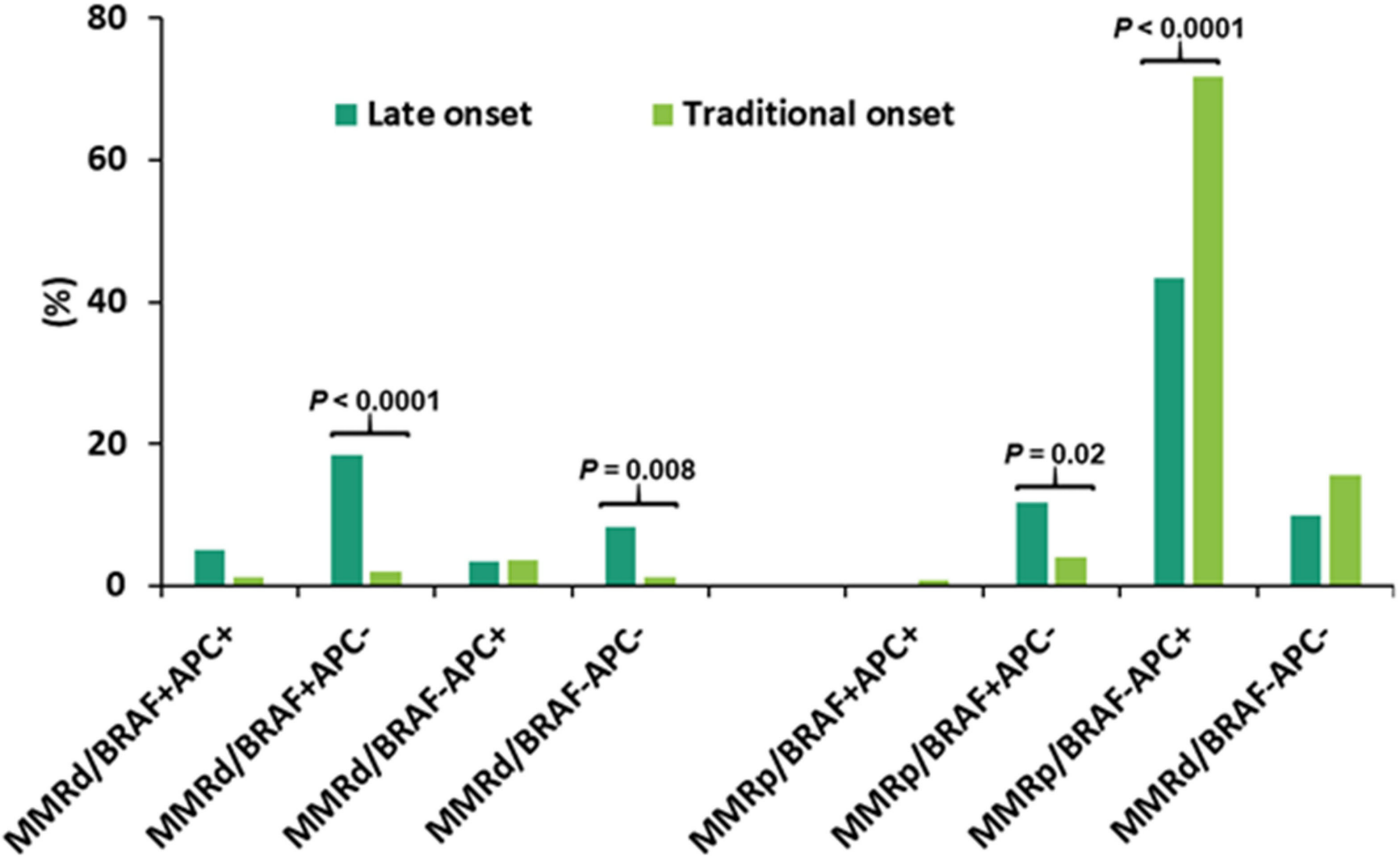
Categorization of CRC according to mismatch repair (MMR) status, *BRAF* p.V600E and *APC* mutations in late-onset CRC (≥80y, n = 60) and traditional-onset CRC (50-69y, n = 251). Late-onset CRC showed a significantly higher incidence of dMMR/*BRAF*+*APC*-, dMMR/*BRAF*-*APC*- and pMMR/*BRAF*+*APC*- and a significantly lower incidence of pMMR/*BRAF*-*APC*+. dMMR: mismatch repair deficiency; pMMR: mismatch repair proficiency, *BRA*F+: *BRAF* p.V600E mutant, *BRAF*-: *BRAF* p.V600E wild type, *APC*+: *APC* mutant, *APC*-: *APC* wild type.

CRCs were segregated into 8 subgroups according to MMR deficiency, *BRAF* p.V600E and *APC* mutations. The dominant population was pMMR CRC with wild-type *BRA*F and mutant *APC* (pMMR/*BRAF* ^-^ *APC* ^+^) (67%), followed by pMMR/*BRAF* ^-^ *APC* ^-^ CRC (15%). Advanced age was significantly associated with a higher rate of *BRAF* ^+^ *APC* ^-^ in both dMMR CRC and pMMR CRC, a lower rate of pMMR/*BRAF* ^-^ *APC* ^+^ CRC, and a trend of a higher rate of dMMR/*BRAF* ^-^ *APC* ^-^ CRC (**Table 3**). When the late-onset CRC (≥ 80y) was compared to the traditional-onset CRC (50-69y), there was also a significantly higher rate of dMMR/*BRAF* ^+^ *APC* ^-^ CRC (18% vs. 2.0%), dMMR/*BRAF* ^-^ *APC* ^-^ CRC (8.3% vs. 1.2%) and pMMR/*BRAF* ^+^ *APC* ^-^ CRC (12% vs. 4.0%) and a significantly lower incidence of pMMR/*BRAF* ^-^ *APC* ^+^ CRC (43% vs. 72%) (**Figure 5**).

**Table 3:** Association of advanced age with CRCs categorized by mismatch repair deficiency, *BRAF* p.V600E mutation and *APC* mutations.

	Total	>80 y^a^	70-79 y^a^	60-69 y^a^	50-59 y^a^	40-49 y^a^	<40 y^a^	
	(n=544)	(n=60)	(n=109)	(n=132)	(n=119)	(n=85)	(n=39)	*P-*value
dMMR	73 (13%)	21 (35%)	18 (17%)	8 (6.1%)	12 (10%)	9 (11%)	5 (13%)	
*BRAF* ^+^ *APC* ^+^	8 (1.5%)	3 (5.0%)	2 (1.8%)	1 (0.8%)	2 (1.7%)	0	0	0.18
*BRAF* ^+^ *APC* ^-^	27 (5.0%)	11 (18%)	10 (9.2%)	4 (3.0%)	1 (0.8%)	1 (1.2%)	0	<0.001
*BRAF* ^-^ *APC* ^+^	24 (4.4%)	2 (3.3%)	3 (2.8%)	2 (1.5%)	7 (5.9%)	6 (7.1%)	4 (10%)	0.12
*BRAF* ^-^ *APC* ^-^	14 (2.6%)	5 (8.3%)	3 (2.8%)	1 (0.8%)	2 (1.7%)	2 (2.4%)	1 (2.6%)	0.07
pMMR	471 (87%)	39 (65%)	91 (83%)	124 (94%)	107 (90%)	76 (89%)	34 (87%)	
*BRAF* ^+^ *APC* ^+^	3 (0.6%)	0	0	1 (0.8%)	1 (0.8%)	0	1 (2.6%)	0.47
*BRAF* ^+^ *APC* ^-^	24 (4.5%)	7 (12%)	6 (5.5%)	5 (3.8%)	5 (4.2%)	1 (1.2%)	0	0.04
*BRAF* ^-^ *APC* ^+^	363 (67%)	26 (43%)	70 (64%)	93 (70%)	87 (73%)	60 (71%)	27 (69%)	0.002
*BRAF* ^-^ *APC* ^-^	81 (15%)	6 (10%)	15 (14%)	25 (19%)	14 (12%)	15 (18%)	6 (15%)	0.5

BRAF^+^, BRAF p.V600E mutation; dMMR, mismatch repair deficiency; pMMR, mismatch repair proficiency.

a At diagnosis of colorectal cancers.

### Multivariate analyses by clinically significant features

Since disease stage at diagnosis and primary location of tumors were significantly associated with advanced age (**Table 1**), we next performed multivariate analysis as an exploratory investigation to understand how molecular features were influenced by age across the entire cohort when controlling for these clinical features (**Table 4**). When compared to younger patients with similar disease stage and primary location, older patients were less likely to have *APC* or *KRAS* mutations but more likely to have *BRAF* p.V600E. (**Table 4A**; per decade of age increase, odds ratio [OR]= 0.70 [95% confidence interval [CI]: 0.60-0.82] for *APC* ^+^; OR=0.86 [95% CI: 0.76-0.98] for *KRAS* ^+^; OR = 1.70 [95% CI: 1.34-2.16] for *BRAF* ^+^). In addition to BRAF p.V600E (OR=4.76; 95% CI: [2.35-9.67], P < 0.001), right-sided CRC also harbored a higher incidence of KRAS mutations (OR=1.41; 95% CI: [0.96-2.08], P = 0.08) and PIK3CA mutations (OR=2.18; 95% CI: [1.41-3.39], P < 0.001).

Table 4AMultivariable analyses adjusted by significant clinical features for association of aging in mutational profiling (*APC*, *KRAS*, *BRAF* and *PIK3CA* mutations).

**Table:** 

Variables	*APC*+		*KRAS*+		*BRAF* p.V600E		*PIK3CA*+	
	OR		OR		OR		OR	
	(95% CI)	*P*-value	(95% CI)	*P*-value	(95% CI)	*P*-value	(95% CI)	*P*-value
Age								
Per decade	0.7		0.86		1.7		1.08	
	(0.60-0.82)	<0.001	(0.76-0.98)	0.03	(1.34-2.16)	<0.001	(0.93-1.25)	0.33
Stage								
1-2	1		1		1		1	
3-4	0.56		1		1.38		0.81	
	(0.36-0.89)	0.01	(0.68-1.47)	1	(0.74-2.59)	0.31	(0.52-1.24)	0.33
Side								
Left	1		1		1		1	
Right	0.74		1.41		4.76		2.18	
	(0.48-1.15)	0.18	(0.96-2.08)	0.08	(2.35-9.67)	<0.001	(1.41-3.39)	<0.001

OR, odds ratio; CI, confidence interval.

OR > 1 indicated the group (or per unit increase) was more likely to be mutation positive compared to reference group.

**Table 4B:** Multivariable analyses adjusted by significant clinical features and stratified by gender for association of aging in mutational profiling dMMR and TP53 mutation.

Variables	dMMR				TP53^+^			
	Male		Female		Male		Female	
	OR		OR		OR		OR	
	(95% CI)	*P*-value	(95% CI)	*P*-value	(95% CI)	*P*-value	(95% CI)	*P*-value
Age								
Per decade	nonlinearity		nonlinearity		1.08		0.83	
increase					(0.88-1.32)	0.45	(0.68-1.00)	0.055
≤ 49	1		1					
50-69	0.37		0.08					
	(0.12-1.08)	0.07	(0.22-2.86)	0.73				
≥ 70	0.23		3.17					
	(0.07-0.81)	0.02	(0.95-10.52)	0.06				
Stage								
1-2	1		1		1		1	
3-4	0.53		0.43		1.37		1.26	
	(0.20-1.38)	0.19	(0.20-0.90)	0.03	(0.76-2.46)	0.29	(0.73-2.19)	0.41
Side								
Left	1		1		1		1	
Right	7.86		5.92		0.43		0.56	
	(2.63-23.46)	<0.001	(2.41-14.56)	<0.001	(0.24-0.76)	0.004	(0.32-0.99)	0.04

dMMR, mismatch repair deficiency; OR, odds ratio; CI, confidence interval.

OR > 1 indicated the group (or per unit increase) was more likely to be dMMR/mutation positive compared to reference group.

Since age-related increased incidence of dMMR and decreased incidence of *TP53* mutations were only seen in female patients (**Figure 2**), gender was also included for analyses (**Table 4B**). The relationship between dMMR incidence and patient age differed between male and female patients. In male patients, older patients with CRC had a lower rate of dMMR. Specifically, older male patients with age of diagnosis ≥70 showed a 77% (95% CI: 19-93%) lower rate of dMMR when compared to younger male patients with similar disease stage and side of colon. By contrast, older female patients ≥70 had a higher rate of dMMR disease when compared to younger female patients age <50 with similar disease stage and side of colon (**Table 4B**; OR=3.17; 95% CI: [0.95-10.52], *P* = 0.06). In female patients, there was a trend toward a lower rate of *TP53* mutated disease in older patients compared to younger patients with similar disease stage and colon side (**Table 4B**; per decade age increase, OR=0.83, 95% CI [0.68-1.00], *P* = 0.055). This relationship was not observed in male patients. The incidence of TP53 mutations was significantly lower in both male (OR=0.43; 95% CI: [0.24-0.76], P = 0.004) and female (OR=0.56; 95% CI: [0.32-0.99], P = 0.004) right-sided CRC.

## Discussion

This investigation adds to a growing body of literature suggesting age-dependent shifts in the clinical and molecular features of CRC (4, 6, 7). The current cohort demonstrated that CRC diagnosed in patients who were 80 years or older (late-onset CRC), as compared to the traditional-onset CRC diagnosed at 50-69 years, had a substantially higher rate of dMMR disease (35%) and *BRAF* p.V600E mutation (35%) and increased incidences of other clinicopathological features associated with MMR deficiency. These included female gender, right-sided primary, high-grade histomorphology and absence of systemic metastasis at diagnosis which confirms findings noted by other groups (7, 8, 21, 22). Given the low number (n=10) of African-American patients in our age 80 or older cohort we did not evaluate the influence of this variable on tumor-specific features. The association of aging with a higher rate of MMR deficiency (*P* = 4x10^-6^) and *BRAF* p.V600E mutation (*P* = 2x10^-9^) were also supported by stratifying patients into 6 groups based on the age. While the higher rate of *BRAF* p.V600E mutations was seen in both male and female elderly patients, the age-related higher rate of dMMR disease was seen exclusively in female patients. BRAF pV600E disease, as previously reported, was more common in cancer of the right colon, suggesting that right and left-sided colonic epithelium produce distinct CRC molecular phenotypes (7, 8). In addition to the findings of MMR deficiency and BRAF p.600E mutations, multivariate analyses also demonstrated a decrease of APC and KRAS mutations with aging and a higher incidence of KRAS and PIK3CA mutations and a low incidence of TP53 mutations in the right-sided CRC, consistent with the previous reports (23–25).

The current study confirmed a positive correlation between *BRAF* p.V600E mutation and dMMR CRC shown in the previous studies (7, 8, 20, 26). In addition, the proportion of dMMR tumors carrying *BRAF* p.V600E was significantly enriched with advanced age. In late-onset (≥80y) group and the 70-79y group, two-thirds of dMMR CRC harbored *BRAF* p.V600E. Sporadic MMR deficiency usually arises from epigenetic silencing of the *MLH1* promoter, a global hypermethylation in CpG islands, and is associated with *BRAF* p.V600E mutation (26–29). The findings suggested that the higher incidence of MMR deficiency in late-onset CRC is most likely predominantly driven by hypermethylation of *MLH1* promoter resulting from *BRAF* mutations (26–29).

Approximately 10-15% of CRC are MMR deficient and 3-5% of CRC are associated with Lynch syndrome (30–32). The recommended paradigm for screening of Lynch syndrome is to examine for *BRAF* p.V600E and/or MLH1 promoter methylation to select dMMR CRC for further germline testing (33, 34). In the NGS era, testing tumor tissues by a comprehensive NGS panel to include *BRAF*, *KRAS*, *NRAS*, MMR genes (*MLH1*, *MSH2*, *MSH6*, *PMS2*, *EPCAM*) and a panel of genes associated with other hereditary CRC not only identifies somatic mutations for targeted therapy and prognostication prediction, but also detect double somatic mutations of the MMR genes and potential germline mutations for further confirmation by testing non-neoplastic tissues (34, 35). In this cohort, potential germline mutations were seen in 50% of dMMR/*BRAF* ^-^ CRC, but none of MMRd/*BRAF* ^+^ CRC. Among the dMMR/*BRAF* ^-^ CRC, patients with potential germline mutations were significantly younger (data not shown). However, obtaining germline testing results for confirmation was not feasible in this retrospective study.

The increased incidence of dMMR with advancing age was only statistically significant in female patients within this cohort while male patients showed a similar dMMR incidence in late-onset CRC as in traditional-onset CRC despite both sexes showing an age-related rise in the incidence of *BRAF* p.V600E mutated CRC. The mechanism for this sex-related difference in the incidence of dMMR CRC in older patients is not entirely clear but it is notable that prior investigations with hormone replacement therapy (HRT) observed a reduction in CRC risk in those that received estrogen replacement (36). The shift in the molecular profile of CRC that develops post-HRT differs between cohorts but a case-control study that stratified patients by age noted female patients age 71 or greater who had previously been on HRT saw the greatest reductions in CRC incidence specifically for microsatellite unstable and/or *BRAF* mutant disease (36, 37). Given the previously established role for estrogen in modifying the epigenetic landscape of cells through regulation of DNA methyltransferases it is possible that menopause-related hormone changes might facilitate *BRAF* pV600E associated *MLH1* silencing in female patients (38). In this retrospective study, however, obtaining the hormone replacement therapy history in a high proportion of patients was not feasible. Further prospective studies are warranted to elucidate the effect of hormone replacement therapy on colorectal tumorigenesis.

The co-association of *BRAF* p.V600E mutation, dMMR disease, and wild-type *APC* may make it hard to decipher if one or several of these factors is responsible for the increased proportion of tumors with these features in late-onset CRC. To delineate these relationships, the proportion of patients with and without each variable was evaluated, and showed association of *BRAF* p.V600E with MMR deficiency was independent of *APC* mutational status and association of *BRAF* p.V600E with wild-type *APC* is independent of MMR deficiency. *APC* mutations and *BRAF* p.V600E were essentially mutually exclusive in pMMR CRC as reported previously, though 11% dMMR CRC harbored concurrent *APC* and *BRAF* p.V600E mutations (39–41). Further categorization of CRC according to these 3 genomic alterations revealed a significantly higher rate of dMMR/*BRAF* ^+^ *APC* ^-^ in late-onset CRC (18% vs. 2.0% in traditional-onset CRC), supporting *MLH1* promoter hypermutation driven by *BRAF* p.V600E as the predominant mechanism of the increased rate of dMMR disease in late-onset CRC. However, the incidences of dMMR/*BRAF* ^-^ *APC* ^-^ (8.3% vs. 1.2%) and pMMR/*BRAF* ^+^ *APC* ^-^ (12% vs. 4.0%) were also significantly higher in the late-onset CRC as compared to traditional-onset CRC, suggesting additional mechanisms independently contribute to a higher rate of MMR deficiency and *BRAF* p.V600E mutation in late-onset CRC.

Testing for *KRAS*, *NRAS* and *BRAF* p.V600E mutations as well as MMR deficiency are recommended as standard-of-care for CRC (12, 19). *BRAF* p.V600E is tested not only for prognostic stratification and evaluation of risk of Lynch syndrome, but also to allow for treatment with recently approved targeted therapy (12, 19). *BRAF* p.V600E, as a prognostic marker, is associated with poor outcomes in CRC (42, 43). Lynch syndrome occurs in approximately only 1% of *BRAF* p.V600E mutated CRC. The NCCN recommended germline testing in *BRAF* p.V600E mutated CRC only for young patients and patients with a significant family history (44). In 2021, the Food and Drug Administration (FDA) in the United States approved combined BRAF inhibitor (encorafenib) plus anti-EGFR monoclonal antibody (cetuximab) for *BRAF* p.V600E mutated metastatic CRC after prior therapy **(** 45, 46 **).** *KRAS* and *NRAS* mutations are predictive for a lack of benefit to epidermal growth factor receptor (EGFR) inhibitor therapy (47, 48).

MMR deficiency is a molecular marker for screening for Lynch syndrome, prognostic stratification and prediction of immune checkpoint therapy efficacy (12, 21, 22). Prior to identification of its therapeutic implications, MMR testing was primarily focused on the identification of patients with Lynch Syndrome in younger patient (<50 years) according to the revised Bethesda guideline in 2004, which was extended to <70 years in 2014 guideline from the National Comprehensive Cancer Network (NCCN) (49, 50). This initial emphasis on screening younger patients for Lynch syndrome may be part of the reason for reduced MMR evaluation in patients diagnosed at 80 year or older as seen in the current cohort. Before the era of targeted therapy and immune checkpoint therapy, *BRAF* p.V600E was associated with inferior outcomes while MMR deficiency was associated with superior outcomes (20, 43, 51, 52). Stratification by *BRAF* p.V600E and MMR status showed the worst prognosis in pMMR/*BRAF* ^+^ patients and the best prognosis in dMMR/*BRAF* ^-^ patients, though results from different studies were not completely consistent (20, 43, 51, 52).

In 2017, the FDA granted approval of immune checkpoint therapy (pembrolizumab) in pediatric and adult patients with dMMR solid tumors agnostic of tumor type (53–55). This included dMMR CRC that has progressed following treatment with fluoropyrimidine, oxaliplatin, and irinotecan. Recent clinical trials, including phase 3 studies, have shown improved survival rates with immune checkpoint therapy as the first-line treatment for dMMR metastatic CRC, which was independent of *BRAF* p.V600E mutation status (56–58). In 2020, pembrolizumab was approved by the FDA for the first-line treatment of patients with unresectable or metastatic dMMR CRC (57). The presence of a *BRAF* pV600E mutation does not appear to impact the responsiveness of these tumors to immune checkpoint therapy (56, 57). These recent advances of targeted therapy for *BRAF* p.V600E mutated CRC and immune checkpoint therapy for dMMR CRC highlight the importance of molecular testing, especially in late-onset CRC which harbors the highest incidence of *BRAF* p.V600E mutation and MMR deficiency.

The increase in dMMR disease is particularly interesting given the higher prevalence of co-morbidities in this older age group leading to an elevated risk of complications from surgical interventions. Recent investigations suggest localized dMMR CRC responds well to immunotherapy which obviated the need for surgery in a small cohort of locally advanced rectal cancer patients recently published by Cercek et al. (59, 60) Given the high rate of dMMR disease in patients ≥80 years old, clinical trials investigating the use of immunotherapy as an alternative to surgery in high-risk older patients may be warranted.

This retrospective cohort has several important limitations that must be considered when interpreting the outlined results. Foremost, this is a retrospective study of patients that underwent NGS and dMMR testing at JHH. While this represents a diverse group of patients across age groups and ethnicities, there may be important unrealized factors that skew the prevalence of different genomic alterations between this cohort and other patient populations such as those that did not undergo NGS and/or dMMR testing. In addition, most patients have their examination of MMR status by either IHC stains or MSI assay in this retrospective study. Although this could lead to a potential bias, IHC and MSI assays is expected to be highly concordant in CRC (21). Furthermore our number of patients over 80 was only 60 patients (20 males and 40 females) making our conclusions hypothesis generating rather than confirmatory. It is notable that a statistically higher rate of patients with late-onset CRC (≥80y) than younger age groups with NGS did not undergo dMMR testing. It is possible that a lower percentage of these patients would have been dMMR than the patients that were examined although it is notable that 5 of the 9 were wild type *APC* and 3 were *BRAF* p.V600E which both co-segregated with dMMR in our dataset.

In this retrospective study, we report on 544 cases of CRC with a particular focus on how the clinical and molecular features of these tumors shift with patient age at diagnosis. These investigations identified a higher rate of *BRAF* p.V600E mutated CRC in older patients along with a higher rate of dMMR disease specifically in female patients. These findings provide important insight into the biology of cancer in older patients in particular in those 80 years and older. Further studies with larger cohorts are needed to elucidate the genomic landscape of late-onset CRC. Prospective studies with treatment plans designed specifically for these older patients are also warranted to evaluate whether this improves clinical outcomes.

## Data availability statement

The raw data supporting the conclusions of this article will be made available by the authors, without undue reservation.

## Ethics statement

The studies involving human participants were reviewed and approved by Johns Hopkins University IRB. Written informed consent for participation was not required for this study in accordance with the national legislation and the institutional requirements.

## Author contributions

EC, DL, EJ, and M-TL contributed to conception and design of the study. EC and M-TL organized the database. EC, H-LT, and M-TL performed the statistical analysis. EC, H-LT, DL, EJ, JD, RX, CG, JE, M-TL provided input of manuscript writing, manuscript revision, read, and approved the submitted version.
